# Synthesis and Evaluation of Biological Activities of Aziridine Derivatives of Urea and Thiourea

**DOI:** 10.3390/molecules23010045

**Published:** 2017-12-25

**Authors:** Aleksandra Kowalczyk, Adam M. Pieczonka, Michał Rachwalski, Stanisław Leśniak, Paweł Stączek

**Affiliations:** 1 Department of Microbial Genetics, Faculty of Biology and Environmental Protection, University of Lodz, Banacha 12/16, 90-237 Lodz, Poland; aleksandra.strzelczyk@biol.uni.lodz.pl; 2 Department of Organic and Applied Chemistry, Faculty of Chemistry, University of Lodz, Tamka 12, 91-403 Lodz, Poland; mrach14@wp.pl (M.R.); slesniak@chemia.uni.lodz.pl (S.L.)

**Keywords:** aziridines, thiourea derivatives, antimicrobial activity, cytotoxicity

## Abstract

In the present paper, we report the synthesis and evaluation of in vitro antimicrobial activities of aziridine-thiourea derivatives. A series of aziridines in reaction with isocyanates and isothiocyanates to obtain urea and thiourea derivatives were used. The structures of all new products were confirmed based on spectroscopic data (^1^H-NMR, ^13^C-NMR, HR-MS). These compounds were screened for their in vitro antimicrobial activity against a panel of Gram-positive and Gram-negative strains of bacteria. Six of the tested compounds appeared to be promising agents against reference strains of *Escherichia coli*, *Staphylococcus aureus* and *Staphylococcus epidermidis*. Subsequently, compounds exhibiting promising antibacterial activity were tested against twelve clinical isolates of *S. aureus* from three different sources of infection. The most bactericidal compounds (MIC = 16–32 µg/mL) showed better antibacterial activity against MRSA than ampicillin and streptomycin. The in vitro cytotoxicity analysis on L929 murine fibroblast and HeLa human tumor cell line using the MTT assay allowed us to select the least toxic compounds for future investigation.

## 1. Introduction

Aziridines are nitrogen-containing, three-membered ring heterocycles, which are widely known as useful reactive intermediates in the synthesis of amino acid derivatives, azomethine ylides or chiral amino alcohols [1,2,3]. In addition, they are used as chiral auxiliaries and chiral ligands in asymmetric synthesis [4,5,6,7,8] or in fused heterocycles [9]. Besides their importance as reactive intermediates, aziridine-containing compounds possess many biological activities especially antitumor and antibacterial ones, due to the presence of the aziridine ring [10]. Aziridines are powerful alkylating agents and their in vivo potency is based primarily on toxicity rather than specific activity. The toxicity of aziridine derivatives depends on their structure, and several important natural products, such as mitomycin C [11], porfiromycin [12], and carzinophilin A [13] are well known in the literature as biologically active agents. Physiological effect of mitomycin C relies on aziridine ring opening and interaction with guanine nucleobase of DNA in the alkylation reaction. This leads to covalent interstrand DNA–DNA crosslink formation, inhibition of replication and finally to cell death. Aziridines with the amide function are currently of special interest with Imexon as the well-known representative. Imexon is an anticancer agent active especially against human myeloma cells where it binds to cellular thiols, reduces the amount of glutathione and cysteine in target cells which leads to elevated levels of reactive oxygen species (ROS). As a result, mitochondria swell, cytochrome c is released, caspase 3 and 9 are activated and the cells enter an apoptotic pathway [14,15]. Imexon is also known to disrupt the redox balance of the endoplasmic reticulum which inhibits protein translation and arrests cell growth [16]. In combination with docetaxel it has been successfully applied in the trial treatment of different cancers [17]. Other work related its activity to suppression of B-lymphocyte activation which suggested Imexon to be useful in the treatment of B-cell or plasma cell lymphomas or neoplasias, certain autoimmune disorders and infection with Rauscher leukemia virus [18,19]. Natural aziridine alkaloids, as well as their lipophilic semi-synthetic, and synthetic analogs, in addition to antitumor activity, have also a strong antibacterial activity [20]. Well known mitomycin A, C and mitosane compounds show antimicrobial activity primarily against Gram-positive bacteria and *Klebsiella pneumoniae* [10,20]. Azirinomycin (3-methyl-2*H*-azirine-2-carboxylic acid) is most active against *Staphylococcus aureus* followed by *Streptococcus faecalis*, *Proteus vulgaris* and *Bacillus subtilis*. The methyl ester of azirinomycin exhibited broad spectrum antibiotic activity in vitro against both Gram-positive and Gram-negative bacteria [21,22]. The alkaloidal antibiotic ficellomycin produced by *Streptomyces ficellus* inhibited growth of Gram-positive bacteria in vitro and in vivo during treatment of experimental *Staphylococcus aureus* infections in mice [23]. Some naturally occurring peptides containing an aziridine ring, for example madurastatin A1 and B1, consisting of Ser and salicylic acid moieties exhibit antibacterial activity against *Micrococcus luteus* [24]. Anticancer drugs, azinomycin A and B are active against both Gram-positive and Gram-negative bacteria. Two other aziridine derivatives, azicemicin A and B demonstrate strong antibacterial activity mainly against *Mycobacterium smegmatis*, *Escherichia coli* NIHJ, *Corynebacterium bovis* and *Micrococcus luteus* [25]. A chromoprotein antitumor drug maduropeptin exhibits inhibitory activity against Gram-positive bacteria [26]. There are also known some aziridine, 2-aminoethylaziridine and azirine complexes of copper(II) and palladium(II) with potent antimicrobial properties against Gram-positive bacteria (*S. aureus*, *S. epidermidis*, *E. faecalis*) [27]. Moreover, one derivative of diaziridinyl quinone isoxazole hybrid showed good antibacterial and anti-biofilm activities with very low MIC values against *S. aureus* and *B. subtilis* and it also exhibited antifungal activity against *Candida albicans* [28].

Many other natural or synthesized compounds with different structures like lipids, steroids, amino acids and peptides containing the aziridine moiety have also shown biological activity and are promising candidates for the development of new drugs against several diseases [10,27,29,30].

In the present paper, we focused on the design and synthesis of structurally novel urea and thiourea aziridine derivatives and evaluation of their biological activity based on the fact that many aziridine ring containing compounds have demonstrated antibacterial and cytotoxic activities.

## 2. Results and Discussion

### 2.1. Chemistry

**Figure 1:**
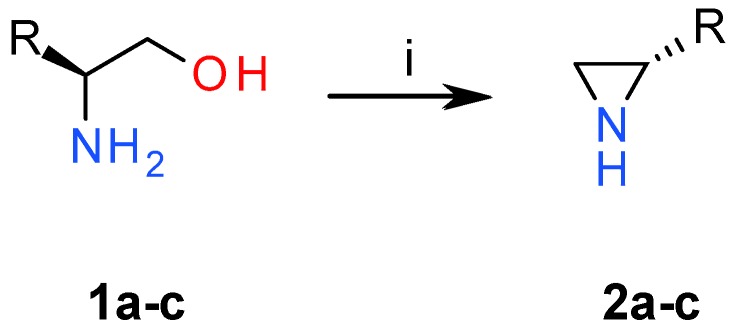
*Reagents and conditions:* (i) 1. ClSO_3_H, MeCN; 2. 5 M NaOH, reflux.

In continuation of the studies on the synthesis and application of chiral aziridines [1,8,10,29,31,32,33], we used chiral aminoalcohols **1** to convert them into NH aziridines **2** via a modified Wenker synthesis [34]. Aminoalcohols **1a**–**b** in the presence of chlorosulfonic acid form sulfonic esters under mild conditions in quantitative yields. These esters under strong basic conditions were converted into the corresponding aziridines **2a**–**c** at elevated temperature (Figure 1).

Non-protected aziridines are stable compounds under basic conditions and they can easily react with diverse electrophiles. In our case, we used a series of aziridines **2** in reactions with isothiocyanates to obtain urea and thiourea derivatives. This methodology was previously used in 1987 to obtain mitomycin C derivatives [35], but none of the presented compounds exhibited significant activity against leukemia cells. 

**Figure 2:**
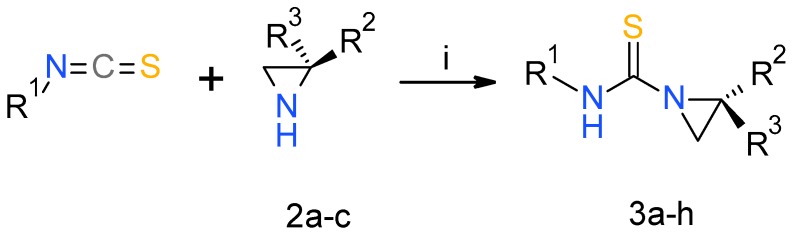
*Reagents and conditions*: (i) CH_2_Cl_2_, 30 min to 16 h.

In the course of our studies, three aziridines **2a**–**c** bearing different substituents ((*S*)-2-methyl, (*S*)-2-isopropyl, 2,2-dimethyl), were reacted with a series of isothiocyanates yielding expected products in high yields after 16 h (Figure 2, Table 1) [30].

Structures of all new products were confirmed based on spectroscopic data (^1^H-NMR, ^13^C-NMR, HR-MS). For example, the ^1^H-NMR spectrum of thiourea **3a** revealed the presence of two characteristic doublets at 2.17 and 2.51 ppm attributed to CH_2_ group of aziridine ring and broad signal at 6.61 ppm attributed to NH group. In the ^13^C-NMR spectra absorbance of a C=S group was found at 197.9 ppm. Due to the fact that many aziridine alkaloids exhibit good antimicrobial activity against selected phatogens [10], we decided to test preliminary the antibacterial and cytotoxic activity of our aziridine derivatives. Based on the results obtained from biological analysis of **3a**–**3h** compounds, we decided to confirm our preliminary conclusions regarding the biological role of some atoms and conformations in analyzed aziridine-containing agents by designing and synthesizing the next set of compounds. 

In pursuit of the second goal, we synthesized aziridine-thiourea derivatives containing sterically crowded substituents, like benzyl (**3i**, **3j**) and benzhydryl (**3k**, **3l**). We also decided to include additional amine function in the aliphatic chain (compounds **3m**–**3p**) as a modification of compound **3a**. We also replaced sulfur atom in compounds **3a** and **3c** to check the influence of the oxygen atom in the new urea derivatives, and we also synthesized an analog of compound **3o** with (*R*)-isopropylaziridine (Table 2) to test if the configuration of the aziridine subunit had any significance to the biological activity. All new compounds were obtained according to a previously described procedure, only the reaction of (*S*)-2-isopropylaziridine **2b** butylisocyanate and cyclohexylisocyanate gave the corresponding urea derivatives **3r**, **3s** in excellent yields after 30 min. 

### 2.2. Biology

All compounds were tested for their antimicrobial activity against a representative panel of bacteria i.e., *Escherichia coli*, *Pseudomonas aeruginosa*, *Staphylococcus aureus*, *Staphylococcus epidermidis*, *Enterococcus faecalis*, *Proteus vulgaris* and *Proteus mirabilis* using nitrofurantoin, ampicillin and streptomycin as reference drugs. The in vitro antimicrobial activities of the compounds **3a**–**3h** at concentrations ranging from 1 to 512 μg/mL were screened using the microdilution method. The results showed that two compounds **3g**, **3h** were inactive against all tested bacteria in analyzed concentrations. Six compounds, **3a**–**3f**, showed antibacterial activity against Gram-positive strains (MIC value ranging from 16 to 512 μg/mL), however, lower than the reference drugs, and were inactive against *P. aeruginosa* and *Proteus* strains. The in vitro results of antibacterial activity of these compounds are presented in Table 3 as a minimal inhibitory concentration (MIC) and a minimal bactericidal concentration (MBC).

Among Gram-positive species, the most sensitive to all of the active compounds were two strains of *S. aureus* and *S. epidermidis*. The most effective against these strains were compounds **3a**, **3b**, **3c**, **3f** (MIC = 16–32 µg/mL) although their activities were lower than those exhibited by ampicillin and streptomycin which are the antibiotics commonly used in the therapy of staphylococcal infections. Among six tested compounds, **3b** was the only effective agent against *E. coli* reference strain (MIC = 32 µg/mL). **3f** was the most active compound especially against Gram-positive strains with activity similar to nitrofurantoin (MIC = 16 µg/mL), however, in case of *S. aureus* strains, its activity was more bacteriostatic than bactericidal with high MBC value (MBC = 128 µg/mL) while other agents showed bactericidal activity (MBC/MIC = 2 or 3, respectively). Compounds **3d** and **3e** showed lower antibacterial activity than other compounds with MIC= 128–256 µg/mL. Our observations suggest that 2,2-dimethyl substituent at R^2^ position of aziridine significantly reduces antibacterial activity (Table 3, see data **3e** vs. **3b** and **3g** vs. **3c**). The most potent derivatives were aziridines with (*S*)-2-methyl or (*S*)-2-isopropyl moiety at R^2^ however their activity also depended on the type of substituent at R^1^ position. For R^1^-methyl derivatives the replacement of (*S*)-2-methyl with (*S*)-2-isopropyl group highly increased antibacterial activity (4–8 fold) (Table 2, see data **3d** vs. **3b**). On the other hand, for R^1^-cyclohexyl derivatives the replacement of (*S*)-2-methyl with (*S*)-2-isopropyl did not have such significant consequences, however, compound **3f** with (*S*)-2-methyl group showed two-fold stronger antimicrobial activity than **3c**.

Considering good activity of the tested compounds, especially **3a**, **3b**, **3c**, **3f**, against *Staphylococcus* spp., a set of 12 *S. aureus* clinical strains including the ones isolated from two typical sources such as naso-pharynx (carrier state) and ulcers/furuncles (skin and soft tissue infections), but also those from infected bones (invasive infections) were tested against these agents. *S. aureus* D15 and D17 strains were characterized as MRSA due to their resistance to high concentrations of oxacillin. Similarly to the reference *Staphylococcus* spp. Strains, clinical isolates displayed high level of susceptibility to the analyzed compounds (MIC ranging from 16 to 64 µg/mL, Table 4). 

Again, compound **3f** showed the strongest activity, with MIC being equal to 8–16 µg/mL for all of the tested clinical isolates, which for most of the strains was a better result than in case of ampicillin and nitrofurantoin (used as positive control drugs). On the other hand, the other common antibiotics oxacillin and streptomycin were the most active in almost all cases, except for the two MRSA strains isolated from bones (*S. aureus* D15, and D17, which were 16–32 times more sensitive to **3f** compound and 8–16 times to **3a**, **3b**, **3c** compounds. For all four tested compounds, the MBC values were in a similar range, suggesting their bactericidal activity (MBC/MIC = 2).

**Figure 3:**
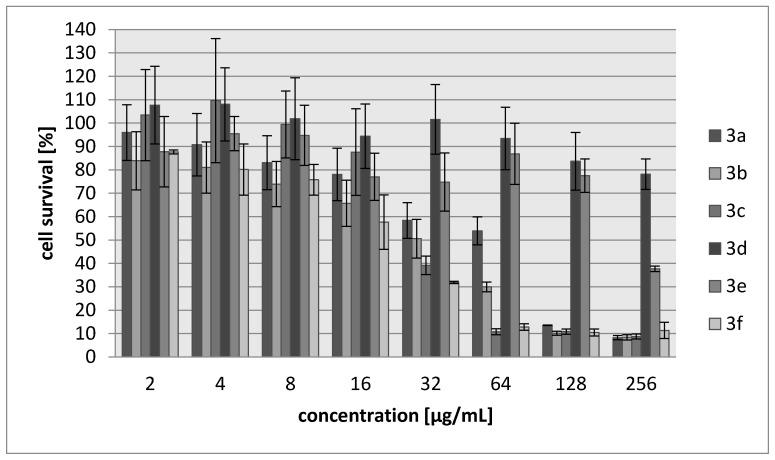
Concentration-dependent cytotoxic activity of **3a**, **3b**, **3c**, **3d**, **3e**, **3f** for L929 cell line.

**Figure 4:**
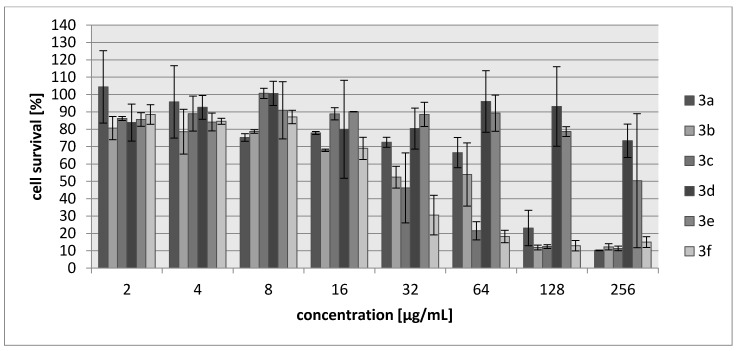
Concentration-dependent cytotoxic activity of **3a**, **3b**, **3c**, **3d**, **3e**, **3f** for HeLa cell line.

Many aziridine-containing compounds are known to demonstrate strong anticancer activity. The cytotoxic activities of **3a**–**3f** were assessed using L929 murine cell line (recommended by the International Standard ISO 10993:2009 for evaluation of cytotoxic activities) as well as HeLa human tumor cell line. The percentage of viability inhibition compared to the negative control in which cells were grown in the absence of tested compounds was estimated for concentrations ranging from 2 to 256 µg/mL of the compound. A common antitumor drug, cisplatin, was used as a positive control. Compounds **3d** and **3e** showed weak cytotoxic effect on both tested cell lines and their activity was ~30-fold lower than for cisplatin (Table 5, Figure 3 and Figure 4). The most toxic were compounds which were the most bactericidal as well (**3b**, **3f**) with IC_30_ = 20–28 µg/mL which is only nearly three-fold lower activity than in case of cisplatin however, they showed no selectivity against tumor cells.

Eleven new compounds, **3i**–**3s**, were tested for their antimicrobial activity against the same reference panel of bacteria and again using nitrofurantoin, ampicillin and streptomycin as reference drugs. The in vitro antimicrobial activities of the compounds **3i**–**3s** at concentrations ranging from 1 to 512 μg/mL were screened using the microdilution method. The results showed that five compounds **3i**, **3n**, **3r**, **3s** and **(*R*)-3o** were completely inactive against all tested bacteria in analyzed concentrations. Interestingly, both urea derivatives had no biological activity compared to their thiourea analogs (Table 2 and Table 6, see data **3r** vs. **3a** and **3s** vs. **3c**) which suggests that the sulphur atom is a principal factor for antibacterial properties. Moreover, an analog of compound **3o** with (*R*)-isopropylaziridine also had no significant biological activity which confirmed that configuration of the aziridine plays an important role for evolving antimicrobial profiles. Six compounds **3j**–**3o** and **3p** showed antibacterial activity mainly against Gram-positive strains (MIC value ranging from 4 to 256 μg/mL) and were inactive against *P. aeruginosa* and *Proteus* strains. The in vitro results of antibacterial activity of these compounds are presented in Table 6 as a minimal inhibitory concentration (MIC). For the two most active compounds **3o** and **3p** minimal bactericidal concentration was also determined (Table 7). For both compounds, the MBC values were in the similar range as MIC, suggesting their bactericidal activity (MBC/MIC = 2 or 3).

Considering good activity of these two compounds against *S. aureus* reference strains, we used a set of 12 *S. aureus* clinical strains to test antibacterial activity of **3o** and **3p**. Our results revealed satisfactory activity only in case of compound **3o** with MIC being equal to 32 µg/mL for all of the tested clinical isolates, which, for most of the strains, was a better result than in case of ampicillin. Moreover, two MRSA strains isolated from bones (*S. aureus* D15, and D17 were 8–16 times more sensitive to **3o** compound. For both tested compounds, the MBC values were in the similar range, suggesting their bactericidal activity (MBC/MIC = 2, Table 8).

**Figure 5:**
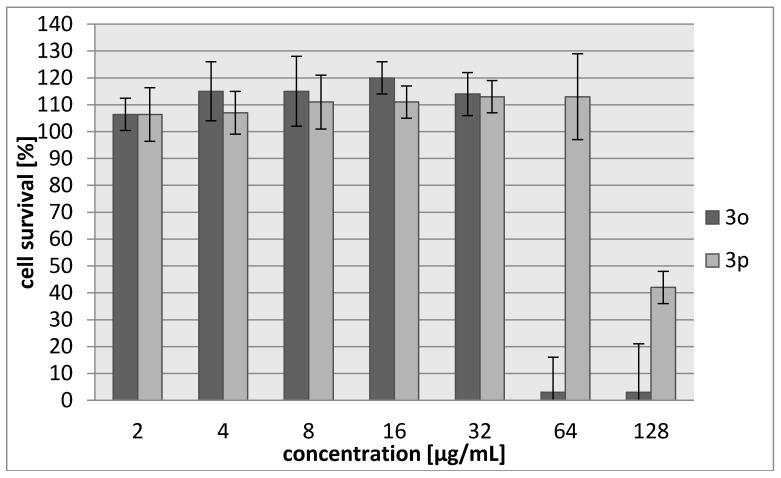
Concentration-dependent cytotoxic activity of **3o** and **3p** for L929 cell line.

**Figure 6:**
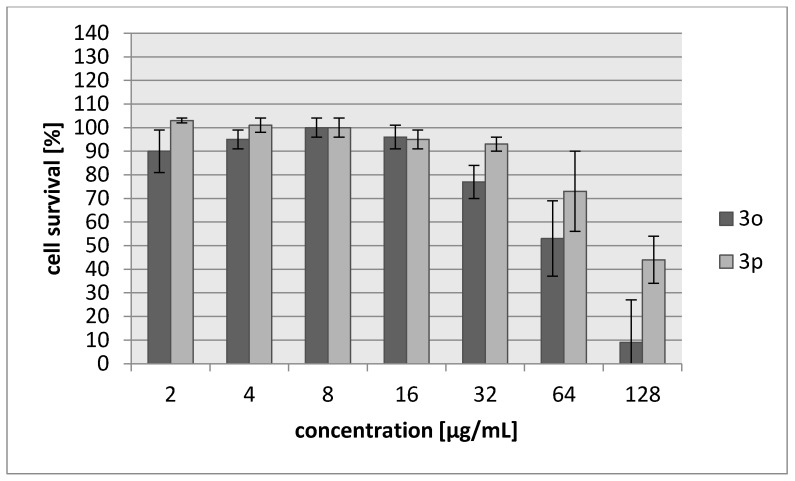
Concentration-dependent cytotoxic activity of **3o** and **3p** for HeLa cell line.

As in the case of previous compounds, the cytotoxic activities of **3o**–**3p** were assessed using L929 murine cell line as well as HeLa human tumor cell line. The percentage of viability inhibition compared to the negative control in which cells were grown in the absence of tested compounds was estimated for concentrations ranging from 2 to 128 µg/mL of the compound. Compound **3o** was more toxic (IC_30_ = 42–45 µg/mL), with cytotoxicity about two-fold higher than **3p** (Table 9, Figure 5 and Figure 6). However, **3o** seemed to be harmless for cells in a concentration corresponding to its antibacterial activity. Both compounds showed no selectivity against tumor cells.

To date, many anticancer agents have been found to show antimicrobial activity against several bacterial pathogens. Among them, mitomycin C as well as other aziridine derivatives have been tested. It is believed that, like in case of their anticancer activity, these compounds act mainly as alkylating agents also in bacterial cells. For example, mitomycin C which was found to be passively transported into the cells, may be used not only for killing metabolically active bacteria but also act against dormant presister cells, leading to formation of DNA crosslinks [36]. However, despite this typical crosslinking activity of aziridine derivatives, other possible modes of antibacterial action cannot be excluded. For example, naturally occurring aziridine derivative (2*S*,3*S*)-aziridine-2,3-dicarboxylic acid was found to inhibit the activity of *E. coli* aspartase [37]. We presume that the mechanism of action of the aziridine derivatives described in this paper may be typical as for the other members of this group of compounds, however, on the basis of this study, it is too early to predict it.

## 3. Experimental Section

### 3.1. Chemistry

General: Melting points were determined in a capillary using a STUART SMP30 and were uncorrected. The ^1^H- (600 MHz), ^13^C{1H}- (150 MHz) spectra were measured on a Bruker Avance III instrument (Bruker, Billerica, MA, USA) using solvent signals as reference. Chemical shifts (δ) are given in ppm and coupling constants *J* in Hz. Assignments of signals in^13^C-NMR spectra were made on the basis of HMQC experiments. HR-MS: Bruker Esquire LC spectrometers (Bruker Daltonics). All solvents are commercially available reagents and were used as received. Aziridines **2a**–**c** were obtained according to a published procedure [34].

### 3.2. General Procedure for Synthesis of Urea and Thiourea Derivatives ***3***

To a stirred solution of an aziridine **2** (1 mmol) in CH_2_Cl_2_ (5 mL) at 20 °C, an equimolar quantity of the isocyanate or isothiocyanate was slowly added. The mixture was stirred for 16 h at room temperature (**3a**–**p**), or 30 min (**3r**, **3s**), the solution was concentrated and the resulting mixture was purified by flash chromatography (SiO_2_/CH_2_Cl_2_).

*(2S)-N-Butyl-2-isopropyl-aziridine-1-carbothioamide* (**3a**): colorless crystals; yield: 92%; m.p. 71–72 °C (MeOH). ^1^H-NMR (600 MHz, CDCl_3_, δ, ppm): 6.61 (1H, br. s, NH); 3.61–3.57 (2H, m, N-CH_2_); 2.51 (1H, d, *J* = 6.6 Hz, CH_2_); 2.31–2.28 (1H, m, aziridine CH); 2.17 (1H, d, *J* = 4.2 Hz, CH_2_); 1.63–1.35 (5H, m); 1.07 (3H, d, *J* = 6.6 Hz, CH_3_); 0.98 (3H, d, *J* = 7.2 Hz, CH_3_); 0.95 (3H, t, *J* = 1.8 Hz, butyl CH_3_). ^13^C-NMR (150 MHz, CDCl_3_, δ, ppm): 197.9 (C=S); 48.9 (aziridine CH); 45.8 (N-CH_2_); 34.8 (aziridine CH_2_); 30.8 (CH); 30.6, 20.1 (2 CH_2_); 20.0, 19.3 (2 CH_3_); 13.7 (butyl CH_3_). HR-EI-MS: 200.1350 (M^+^, C_10_H_20_N_2_S^+^; calcd. 200.1347).

*(2S)-2-Isopropyl-N-methyl-aziridine-1-carbothioamide* (**3b**): colorless crystals; yield: 90%; m.p. 65–66 °C (MeOH). ^1^H-NMR (600 MHz, CDCl_3_, δ, ppm): 6.67 (1H, br. s, NH); 3.16 (3H, d, *J* = 5.4 Hz, N-CH_3_); 2.55 (1H, d, *J* = 6.6 Hz, CH_2_); 2.37–2.33 (1H, m, aziridine CH); 2.21 (1H, d, *J* = 4.2 Hz, CH_2_); 1.62–1.55 (1H, m, isopropyl CH); 1.10 (3H, d, *J* = 6.6 Hz, CH_3_); 1.01 (3H, d, *J* = 7.2 Hz, CH_3_). ^13^C-NMR (150 MHz, CDCl_3_, δ, ppm): 198.2 (C=S); 48.9 (aziridine CH); 34.9 (aziridine CH_2_); 33.2 (N-CH_3_); 30.8 (CH); 20.0, 19.4 (2 CH_3_). HR-EI-MS: 158.0879 (M^+^, C_7_H_14_N_2_S^+^; calcd. 158.0878).

*(2S)-N-Cyclohexyl-2-isopropyl-aziridine-1-carbothioamide* (**3c**): colorless crystals; yield: 89%; m.p. 75–76 °C (MeOH). ^1^H-NMR (600 MHz, CDCl_3_, δ, ppm): 6.60 (1H, br. s, NH); 4.18–4.16 (1H, m, N-CH); 2.49 (1H, d, *J* = 6.6 Hz, aziridine CH_2_); 2.29–2.26 (1H, m, aziridine CH); 2.07 (1H, d, *J* = 4.2 Hz, aziridine CH_2_); 2.06–2.04 (2H, m, cyclohexyl CH_2_); 1.72–1.37 (6H, m); 1.24–1.17 (3H, m); 1.07 (3H, d, *J* = 6.6 Hz, CH_3_); 0.98 (3H, d, *J* = 7.2 Hz, CH_3_). ^13^C-NMR (150 MHz, CDCl_3_, δ, ppm): 197.7 (C=S); 54.1 (cyclohexyl CH); 48.9 (aziridine CH); 34.9 (aziridine CH_2_); 33.6, 33.4, 25.6, 25.5, 24.7 (5 CH_2_); 30.9 (CH); 20.0, 19.3 (2 CH_3_). HR-EI-MS: 226.1500 (M^+^, C_12_H_22_N_2_S^+^; calcd. 226.1504).

*(2S)-N-Methyl-2-methyl-aziridine-1-carbothioamide* (**3d**): colorless crystals; yield: 89%; m.p. 70–71 °C (MeOH). ^1^H-NMR (600 MHz, CDCl_3_, δ, ppm): 6.81 (1H, br. s, NH); 3.07 (3H, s, N-CH_3_); 2.54–2.52 (1H, m, CH); 2.46 (1H, d, *J* = 6.6 Hz, CH_2_); 2.07 (1H, d, *J* = 4.2 Hz, CH_2_); 1.25 (3H, d, *J* = 5.4 Hz, aziridine CH_3_). ^13^C-NMR (150 MHz, CDCl_3_, δ, ppm): 198.5 (C=S); 38.2 (aziridine CH); 37.0 (aziridine CH_2_); 33.2 (N-CH_3_); 17.6 (aziridine CH_3_). HR-EI-MS: 130.0569 (M^+^, C_5_H_10_N_2_S^+^; calcd. 130.0565).

*(2S)-N-Methyl-2,2-dimethyl-aziridine-1-carbothioamide* (**3e**): colorless crystals; yield: 94%; m.p. 77–78 °C (MeOH). ^1^H-NMR (600 MHz, CDCl_3_, δ, ppm): 6.39 (1H, br. s, NH); 3.10 (3H, d, *J* = 5.4 Hz, N-CH_3_); 2.40 (2H, s, CH_2_); 1.23 (6H, s, 2 aziridine CH_3_). ^13^C-NMR (150 MHz, CDCl_3_, δ, ppm): 195.8 (C=S); 42.5 (aziridine CH_2_); 33.2 (N-CH_3_); 22.4 (aziridine CH_3_). HR-EI-MS: 144.0722 (M^+^, C_6_H_12_N_2_S^+^; calcd. 144.0721).

*(2S)-N-Cyclohexyl-2-methyl-aziridine-1-carbothioamide* (**3f**): colorless crystals; yield: 97%; m.p. 68–67 °C (MeOH). ^1^H-NMR (600 MHz, CDCl_3_, δ, ppm): 6.52 (1H, br. s, NH); 4.12–4.08 (1H, m, N-CH); 2.54–2.51 (1H, m, aziridine CH); 2.42 (1H, d, *J* = 6.6 Hz, aziridine CH_2_); 2.07 (1H, d, *J* = 4.2 Hz, aziridine CH_2_); 2.06–1.20 (10H, m); 1.25 (3H, d, *J* = 6.0 Hz, CH_3_). ^13^C-NMR (150 MHz, CDCl_3_, δ, ppm): 198.7 (C=S); 54.2 (cyclohexyl CH); 38.0 (aziridine CH); 36.9 (aziridine CH_2_); 33.6, 33.4, 25.6, 25.5, 24.7 (5 CH_2_); 17.5 (aziridine CH_3_). HR-EI-MS: 198.1198 (M^+^, C_10_H_18_N_2_S^+^; calcd. 198.1191).

*(2S)-N-Cyclohexyl-2,2-dimethyl-aziridine-1-carbothioamide* (**3g**): colorless crystals; yield: 96%; m.p. 76–77 °C (MeOH). ^1^H-NMR (600 MHz, CDCl_3_, δ, ppm): 6.24 (1H, br. s, NH); 4.19–4.13 (1H, m, N-CH); 2.38 (2H, s, aziridine CH_2_); 2.03–1.23 (10H, m); 1.15 (6H, s, 2 CH_3_). ^13^C-NMR (150 MHz, CDCl_3_, δ, ppm): 192.8 (C=S); 54.5 (cyclohexyl CH); 42.1 (aziridine CH_2_); 33.3, 32.5, 25.5, 25.2, 24.8 (5 CH_2_); 22.0 (aziridine CH_3_). HR-EI-MS: 212.1351 (M^+^, C_11_H_20_N_2_S^+^; calcd. 212.1347).

*(2S)-N-Allyl-2-isopropyl-aziridine-1-carbothioamide* (**3h**): colorless crystals; yield: 76%; m.p. 65–66 °C (MeOH). ^1^H-NMR (600 MHz, CDCl_3_, δ, ppm): 6.56 (1H, br. s, NH); 5.86–5.80 (2H, m, CH_2_=); 5.20–5.16 (1H, m, CH=); 4.19–4.17 (allyl CH_2_); 2.46 (1H, d, *J* = 6.6 Hz, CH_2_); 2.28–2.25 (1H, m, CH); 2.14 (1H, d, *J* = 4.2 Hz, CH_2_); 1.53–1.47 (1H, m); 1.01 (3H, d, *J* = 6.6 Hz, CH_3_); 0.92 (3H, d, *J* = 7.2 Hz, CH_3_). ^13^C-NMR (150 MHz, CDCl_3_, δ, ppm): 198.1 (C=S); 132.7 (CH_2_=); 117.6 (CH=); 48.9 (aziridine CH); 48.3 (CH_2_); 34.9 (aziridine CH_2_); 30.8 (isopropyl CH); 20.0, 19.0 (2 aziridine CH_3_). HR-EI-MS: 184.1039 (M^+^, C_9_H_16_N_2_S^+^; calcd. 184.1034).

*(2S)-N-Cyclohexyl-2-isopropyl-aziridine-1-carboxamide* (**3s**): colorless crystals; yield: 96%; m.p. 74–75 °C (MeOH). ^1^H-NMR (600 MHz, CDCl_3_, δ, ppm): 5.16 (1H, br. s, NH); 3.57–3.55 (1H, m, N-CH); 2.30 (1H, d, *J* = 6.6 Hz, aziridine CH_2_); 2.10–2.07 (1H, m, aziridine CH); 1.92–1.85 (2H, m, cyclohexyl CH_2_); 1.82 (1H, d, *J* = 4.2 Hz, aziridine CH_2_); 1.70–1.67, 1.61–1.57 (4H, 2 m, 2 cyclohexyl CH_2_); 1.42–1.31 (3H, m, isopropyl CH, cyclohexyl CH_2_); 1.18–1.12 (2H, m, cyclohexyl CH_2_);1.04 (3H, d, *J* = 6.6 Hz, CH_3_); 0.96 (3H, d, *J* = 7.2 Hz, CH_3_). ^13^C-NMR (150 MHz, CDCl_3_, δ, ppm): 163.1 (C=O); 54.5 (cyclohexyl CH); 45.9 (aziridine CH); 33.6, 33.4, 25.6, 25.5, 24.7 (5 CH_2_); 31.8 (aziridine CH_2_); 30.8 (CH); 20.0, 19.3 (2 CH_3_). HR-EI-MS: 210.1734 (M^+^, C_12_H_22_N_2_O^+^; calcd. 210.1732). 

### 3.3. Biology

#### 3.3.1. Antibacterial Assay

The in vitro antimicrobial activity of newly synthesized compounds was evaluated against the reference strains of Gram-negative (*Escherichia coli* NCTC 8196, *Proteus vulgaris* ATCC 49990, *Proteus mirabilis* ATCC 29906, *Pseudomonas aeruginosa* NCTC 6249), and Gram-positive (*Staphylococcus aureus* ATCC 6538, *Staphylococcus aureus* ATCC 29213, *Enterococcus faecalis* ATCC 29212, *Staphylococcus epidermidis* ATCC 12228) bacterial species. Moreover, the most active (against the reference strains) compounds were examined against a set of twelve clinical isolates of *S. aureus* received from the collection of the Chair of Immunology and Infectious Biology, University of Łódź. These strains were isolated from the following three sources: naso-pharynx of young patients hospitalized at Children’s Hospital in Łódź (*n* = 4), ulcers and furuncles from adult patients of Dermatological Clinic in Łódź (*n* = 4), and from infected bones of patients hospitalized at Oncological Hospital in Łódź (*n* = 4). All strains were kept frozen at −80 °C on Tryptic Soy Broth with 15% of glycerol until testing. Before using, *S. aureus* strains were subcultured on blood agar and identified by routine methods (catalase, coagulase and clumping factor). Minimal inhibitory concentration (MIC) was determined as the lowest concentration of the compound preventing growth of the tested microorganism using microdilution method according to EUCAST guidelines. The *inoculum* density was adjusted to 0.5 McFarland standard. All of the tested compounds were dissolved in dimethyl sulfoxide (DMSO). Concentration of the agents evaluated in Mueller-Hinton broth ranged from 1 to 512 µg/mL. DMSO at the final concentration in the medium had no influence on growth of the tested microorganisms. The incubation was carried out at 37 °C for 18 h and optical density (OD_600_) measurements were determined for bacterial cultures in the presence and absence of the tested compounds. Ampicillin, nitrofurantoin, and streptomycin widely used in the treatment of infectious diseases were used as positive control antimicrobial agents. Minimal bactericidal concentration (MBC), defined as the lowest concentration of a compound that resulted in >99.9% reduction in CFU of the initial *inocula* (2 × 10^8^ cfu) was assessed only for compounds **3c**–**3h** and **3o**, **3p**. MBC was determined by a broth microdilution technique followed by plating out the contents of the wells that showed no visible growth of bacteria onto Mueller-Hinton agar plates and incubating at 35 °C for 18 h. Both MIC and MBC evaluations were performed in triplicates and are given in µg/mL.

#### 3.3.2. Cytotoxicity Assay

Cytotoxic effect of compounds **3c**–**3h** and **3o**, **3p** on host cells was detected by determining cellular viability using MTT reduction assay. Murine fibroblasts L929 cells (ATTC^®^ catalog No. CCL-1, mouse fibroblasts) or human tumor HeLa cells (ATTC^®^ catalog No. CCL-2™, human epithelial cells) were plated in 96-well microplates at density of 1 × 10^4^ cells/mL (100 μL per well) and cultivated in Iscove’s modified Dulbecco’s medium (IMDM), supplemented with 100.0 U/mL penicillin and streptomycin, 5 × 10^−5^ M 2-mercaptoethanol and enriched with 10% fetal bovine serum (FBS). After overnight incubation at 37 °C, the growth medium was removed and 100 μL of medium supplemented with different concentrations of synthetic compounds in the range of 1–300 µg/mL were added. Cells were further incubated for 24 h with tested agents. At the end of the incubation time, the medium was removed and MTT was added to each well at a final concentration of 0.5 mg/mL and plates were incubated for the next 2 h at 37 °C. Then, formazan crystals were solubilized in 150 μL DMSO. The optical density was measured at 550 nm. The results of experiments were shown as mean arithmetic values of eight repeats (two experiments) and the percentage of inhibition of viability compared to control wells was calculated for each concentration of the tested compounds and IC_30_ value (which is considered to be save for tested cells) was determined in each case. The results of the experiments were shown as mean arithmetic values from 3 repeats in each of two independent experiments.

## 4. Conclusions

In conclusion, we have reported the synthesis and preliminary evaluation of the biological activity of 19 novel urea and thiourea aziridine derivatives. While comparing the structure of tested compounds with the antibacterial and cytotoxic activity, we could already draw some substantial conclusions. The presence of sulphur atom is a principal factor for antibacterial properties of aziridine derivatives. The (*S*)-configuration of the aziridine plays an important role for conferring antimicrobial activity. All tested (*S*)-isopropylaziridine derivatives with 1-methyl (**3b**), 1-butyl (**3a**), 1-cyclohexyl (**3c**) and 1-(2-piperidinoethyl) (**3o**) substituents, showed similar satisfactory antibacterial results. The only exception was compound **3h** with a 1-allyl moiety which can suggest that it decreases the antimicrobial activity. We have also observed that a 2,2-dimethyl moiety at C2 position of aziridine (**3e**, **3g**) significantly reduces antibacterial activity. The most bactericidal aziridine derivatives of thiourea also showed the highest toxic activity against both cell lines with no selectivity for tumor cells. It can be explained by the alkylating activity of aziridine ring possessing agents and their in vivo potency based primarily on toxicity rather than specific activity. We selected five of the tested compounds **3a**, **3b**, **3c**, **3f**, **3o** which showed the best activity against clinical *S. aureus* strains, and in two cases of invasive infections of MRSA, these agents exceeded the activity of commonly used antibiotics such as ampicillin, streptomycin and oxacillin by 16-fold. To conclude, all the collected data could provide valuable information for further modifications of these compounds in order to select those with stronger antibacterial activity which would be less harmful. Simultaneously, our attention will be also focused on the improvements leading to the increase of their selectivity against tumor cells, bearing in mind their potential usage in antitumor therapy.

## Figures and Tables

**Table 1:** Yields of the new urea and thiourea derivatives **3a**–**3h**.

Compound	R^1^	R^2^	R^3^	Yield (%)
**3a**	Bu	^i^Pr	H	92
**3b**	Me	^i^Pr	H	90
**3c**	cHex	^i^Pr	H	89
**3d**	Me	Me	H	89
**3e**	Me	Me	Me	94
**3f**	cHex	Me	H	97
**3g**	cHex	Me	Me	96
**3h**	Allyl	^i^Pr	H	76

**Table 2:**
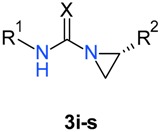
Yields of the urea and thiourea derivatives **3i**–**3s**.

Compound	R^1^	R^2^	X	Yield (%)
**3i**	CH_2_Ph	^i^Pr	S	90
**3j**	CH_2_Ph	Me	S	89
**3k**	CHPh_2_	^i^Pr	S	88
**3l**	CHPh_2_	Me	S	85
**3m**	2-(4-morpholino)ethyl	^i^Pr	S	92
**3n**	2-(4-morpholino)ethyl	Me	S	93
**3o**	2-piperidinoethyl	^i^Pr	S	95
**(*R*)-3o**	2-piperidinoethyl	^i^Pr	S	90
**3p**	2-piperidinoethyl	Me	S	91
**3r**	Bu	^i^Pr	O	96
**3s**	cHex	^i^Pr	O	98

**Table 3:** In vitro antibacterial activity of **3a**, **3b**, **3c**, **3d**, **3e**, **3f** expressed as a minimal inhibitory concentration (MIC) (µg/mL) and a minimal bactericidal concentration (MBC) (µg/mL). AMP: ampicillin, NTF: nitrofurantoin, STR: streptomycin.

Tested Strain	*E. coli* NCTC 8196 NCTC 8196		*S. aureus* ATCC 6538 ATCC 6538		*S. aureus* ATCC 29213 ATCC 29213		*S. epidermidis* ATCC 12228 ATCC 12228	
Compound	MIC	MBC	MIC	MBC	MIC	MBC	MIC	MBC
**3a**	>512	nd	32	128	32	128	32	64
**3b**	32	32	32	64	32	64	16	16
**3c**	>512	nd	32	64	32	64	32	64
**3d**	128	128	256	512	256	512	256	512
**3e**	>512	nd	128	128	128	128	128	128
**3f**	256	512	16	128	16	128	16	16
**NTF**	8	8	16	32	16	32	8	8
**AMP**	4	4	1	1	2	4	1	1
**STR**	1	2	1	1	1	2	>512	>512

Note: nd—not determined.

**Table 4:** In vitro activity of **3a**, **3b**, **3c**, **3f** against clinical isolates of *S. aureus* expressed as the minimal inhibitory concentration (MIC) (µg/mL) and minimal bactericidal concentration (MBC) (µg/mL). OX: oxacillin, AMP: ampicillin, NTF: nitrofurantoin, STR: streptomycin.

Compound	3a		3b		3c		3f		OX		AMP		NTF		STR	
Tested Strain	MIC	MBC	MIC	MBC	MIC	MBC	MIC	MBC	MIC	MBC	MIC	MBC	MIC	MBC	MIC	MBC
	naso-pharynx isolates															
*S. aureus*																
C4	32	64	32	64	32	64	16	16	0.25	0.25	512	512	16	16	8	8
C7	32	64	32	32	32	64	16	16	0.25	0.25	64	128	16	16	8	16
C8	32	64	32	32	32	64	16	16	0.25	0.25	512	>512	16	16	8	16
C19	32	64	32	32	32	64	16	16	0.5	0.5	64	128	32	32	8	8
	ulcers/furuncles isolates															
D12	32	64	16	32	32	128	8	16	0.5	0.5	256	256	32	32	8	16
F1	32	64	32	128	64	128	16	64	0.25	0.25	128	128	32	32	8	8
F7	32	64	32	128	32	64	16	64	0.25	0.25	4	4	16	16	8	16
F12	32	64	16	32	32	64	8	16	0.25	0.25	64	128	16	32	8	32
	bone isolates															
D14	32	64	32	32	32	64	16	16	0.5	0.5	256	512	32	32	8	16
D15 (MRSA)	32	64	32	32	32	64	16	16	>512	>512	>512	>512	32	32	256	>512
D17 (MRSA)	32	64	16	16	32	64	8	32	>512	>512	>512	>512	32	32	256	>512
D20	32	64	32	32	64	128	16	64	0.5	0.5	128	256	16	32	8	8

**Table 5:** Cytotoxic activity data.

Compound	IC_30_ (µg/mL)/(µM) L929 Cells	HeLa Cells
**3a**	68/340	83/388
**3b**	27/171	28/177
**3c**	34/147	69/304
**3d**	>250/>1922	>250/1922
**3e**	214/1485	250/1735
**3f**	20/102	22/111
Cisplatin	7.24/24	6.53/22

**Table 6:** In vitro antibacterial activity of **3j**, **3k**, **3l**, **3m**, **3o**, **3p** expressed as a minimal inhibitory concentration (MIC) (µg/mL). AMP: ampicillin, NTF: nitrofurantoin, STR: streptomycin.

Tested Strain	*E. coli* NCTC 8196 NCTC 8196	*S. aureus* ATCC 6538 ATCC 6538	*S. aureus* ATCC 29213 ATCC 29213	*S. epidermidis* ATCC 12228 ATCC 12228
Compound	MIC (µg/mL)			
**3j**	256	128	128	128
**3k**	256	na*	Na	256
**3l**	256	256	256	256
**3m**	na	128	256	128
**3o**	64	8	8	4
**3p**	256	64	64	64
NTF	8	16	16	8
AMP	4	1	2	1
STR	1	1	1	>512

na*—no activity.

**Table 7:** In vitro antibacterial activity of **3o** and **3p** expressed as a minimal bactericidal concentration (MBC) (µg/mL).

Tested Strain	*E. coli* NCTC 8196 NCTC 8196	*S. aureus* ATCC 6538 ATCC 6538	*S. aureus* ATCC 29213 ATCC 29213	*S. epidermidis* ATCC 12228 ATCC 12228
Compound	MBC (µg/mL)			
**3o**	128	16	16	8
**3p**	256	128	128	128

**Table 8:** In vitro activity of **3o** and **3p** against clinical isolates of *S. aureus* expressed as a minimal inhibitory concentration (MIC) (µg/mL) and a minimal bactericidal concentration (MBC) (µg/mL). OX: oxacillin, AMP: ampicillin, NTF: nitrofurantoin, STR: streptomycin.

Compound	3o		3p		OX		AMP		NTF		STR	
Tested Strain	MIC	MBC	MIC	MBC	MIC	MBC	MIC	MBC	MIC	MBC	MIC	MBC
naso-pharynx isolates												
*S. aureus*												
C4	32	64	256	256	0.25	0.25	512	512	16	16	8	8
C7	32	64	256	256	0.25	0.25	64	128	16	16	8	16
C8	32	64	256	256	0.25	0.25	512	>512	16	16	8	16
C19	32	64	256	256	0.5	0.25	64	128	32	32	8	8
ulcers/furuncles isolates												
D12	32	64	256	256	0.5	0.5	256	256	32	32	8	16
F1	32	64	256	256	0.25	0.25	128	128	32	32	8	8
F7	32	64	256	256	0.25	0.25	4	4	16	16	8	16
F12	32	64	256	256	0.25	0.25	64	128	16	32	8	32
bone isolates												
D14	32	64	256	256	0.5	0.5	256	512	32	32	8	16
D15 (MRSA)	32	64	256	512	>512	>512	>512	>512	32	32	256	>512
D17 (MRSA)	32	64	256	512	>512	>512	>512	>512	32	32	256	>512
D20	32	64	256	512	0.5	0.5	128	256	16	32	8	8

**Table 9:** Cytotoxic activity data.

Compound	IC_30_ (µg/mL)/(µM) L929 Cells	HeLa Cells
**3o**	45/187	42/174
**3p**	103/483	71/333
Cisplatin	7.24/24	6.53/22

## References

[1] Yudin A.K. (2006). Aziridines and Epoxides in Organic Synthesis.

[2] Morieux P., Stables J.P., Kohn H. (2006). Synthesis and anticonvulsant activities of *N*-benzyl (2*R*)-2-acetamido-3-oxysubstituted propionamide derivatives. Bioorg. Med. Chem..

[3] Cimarelli C., Fratoni D., Palmieri G. (2009). A Convenient Synthesis of New Diamine, Amino Alcohol and Aminophosphines Chiral Auxiliaries Based on Limonene Oxide. Tetrahedron Asymmetry.

[4] Leśniak S., Pieczonka A.M., Jarzyński S., Justyna K., Rachwalski M. (2013). ChemInform Abstract: Synthesis and Evaluation of the Catalytic Properties of Semicarbazides Derived from *N*-Triphenylmethyl-aziridine-2-carbohydrazides. Tetrahedron Asymmetry.

[5] Pieczonka A.M., Leśniak S., Rachwalski M. (2014). Direct asymmetric aldol condensation catalyzed by aziridine semicarbazide zinc(II) complexes. Tetrahedron Lett..

[6] Jarzyński S., Leśniak S., Pieczonka A.M., Rachwalski M. (2015). *N*-Trityl-aziridinyl alcohols as highly efficient chiral catalysts in asymmetric additions of organozinc species to aldehydes. Tetrahedron Asymmetry.

[7] Pieczonka A.M., Leśniak S., Jarzyński S., Rachwalski M. (2015). Aziridinylethers as highly enantioselective ligands for the asymmetric addition of organozinc species to carbonyl compounds. Tetrahedron Asymmetry.

[8] Lesniak S., Rachwalski M., Pieczonka A.M. (2014). Optically Pure Aziridinyl Ligands as Useful Catalysts in the Stereocontrolled Synthesis. Curr. Org. Chem..

[9] Cheung L.L.W., Zhi H., Decker S.M., Yudin A.K. (2011). Skeletal Fusion of Small Heterocycles with Amphoteric Molecules. Angew. Chem. Int. Ed..

[10] Ismail F.M.D., Levitsky D.O., Dembitsky V.M. (2009). Aziridine alkaloids as potential therapeutic agents. Eur. J. Med. Chem..

[11] Wakaki S., Marumo H., Tomioka K., Shimizu G., Kato E., Kamada H., Kudo S., Fujimoto Y. (1958). Isolation of new fractions of antitumor mitomycins. Antibiot. Chemother..

[12] Herr R.R., Bergy M.E., Eble T.E., Jahnke H.K. (1960). Porfiromycin, a new antibiotic. Antimicrob. Agents Ann..

[13] Zang H., Gates K.S. (2000). DNA Binding and Alkylation by the “Left Half” of Azinomycin B. Biochemistry.

[14] Dvorakova K., Payne C.M., Tome M.E., Briehl M.M., McClure T., Dorr R.T. (2000). Induction of oxidative stress and apoptosis in myeloma cells by the aziridine-containing agent imexon. Biochem. Pharm..

[15] Dvorakova K., Waltmire C.N., Payne C.M., Tome M.E., Briehl M.M., Dorr R.T. (2001). Induction of mitochondrial changes in myeloma cells by imexon. Blood.

[16] Sheveleva E.V., Landowski T.H., Samulitis B.K., Bartholomeusz G., Powis G., Dorr R.T. (2012). Imexon Induces an Oxidative Endoplasmic Reticulum Stress Response in Pancreatic Cancer Cells. Mol. Cancer Res..

[17] Moulder S., Dhillon N., Ng C., Hong D., Wheler J., Naing A., Tse S., La Paglia A., Dorr R., Hersh E. (2010). A phase I trial of imexon, a pro-oxidant, in combination with docetaxel for the treatment of patients with advanced breast, non-small cell lung and prostate cancer. Investig. New Drugs.

[18] Barr P.M., Miller T.P., Friedberg J.W., Peterson D.R., Baran A.M., Herr M., Spier C.M., Cui H., Roe D.J., Persky D.O. (2014). Phase 2 study of imexon, a prooxidant molecule, in relapsed and refractory B-cell non-Hodgkin lymphoma. Blood.

[19] Herrmann D., Haag R., Bosies E., Bicker U., Kampe W. (1990). Use of Imexone as an Immunosuppressive Agent. Patent.

[20] Kinoshita S., Uzu K., Nakano M., Shimizu A., Takahashi T. (1971). Mitomycin derivatives. 1. Preparation of mitosane and mitosene compounds and their biological activities. J. Med. Chem..

[21] Stapley E.O., Hendlin D., Jackson M., Miller A.K., Hernandez S., Martinez M., Martinez M. (1971). Azirinomycin. I. Microbial production and biological characteristics. J. Antibiot..

[22] Miller T.W., Tristram E.W., Wolf F.J. (1971). Azirinomycin. II. Isolation and chemical characterization as 3-methyl-2(2H) azirinecarboxylic acid. J. Antibiot..

[23] Argoudelis A.D., Reusser F., Whaley H.A. (1976). The Antibiotic U-47,929 and Its Preparation. U.S. Patent.

[24] Harada K.I., Tomita K., Fujii K., Masuda K., Mikami Y., Yazawa K., Komaki H. (2004). Isolation and Structural Characterization of Siderophores, Madurastatins, Produced by a Pathogenic Actinomadura madurae. J. Antibiot..

[25] Tsuchida T., Iinuma H., Kinoshita N., Ikeda T., Sawa T., Hamada M., Takeuchi T. (1995). Azicemicins A and B, a New Antimicrobial Agent Produced by *Amycolatopsis*. J. Antibiot..

[26] Schroeder D.R., Colson K.L., Klohr S.E., Zein N., Langley D.R., Lee M.S., Matson J.A., Doyle T.W. (1994). Isolation, Structure Determination, and Proposed Mechanism of Action for Artifacts of Maduropeptin Chromophore. J. Am. Chem. Soc..

[27] Budzisz E., Bobka R., Hauss A., Roedel J.N., Wirth S., Lorenz I.-P., Rozalska B., Więckowska-Szakiel M., Krajewska U., Rozalski M. (2012). Synthesis, structural characterization, antimicrobial and cytotoxic effects of aziridine, 2-aminoethylaziridine and azirine complexes of copper(II) and palladium(II). Dalton Trans..

[28] Swapnaja K.J.M., Yennam S., Chavali M., Poornachandra Y., Kumar C.G., Muthusamy K., Jayaraman V.B., Arumugam P., Balasubramanian S., Sriram K.K. (2016). Design, synthesis and biological evaluation of diaziridinyl quinone isoxazole hybrids. Eur. J. Med. Chem..

[29] Moonen K., Laureyn I., Stevens C.V. (2004). Synthetic methods for azaheterocyclic phosphonates and their biological activity. Chem. Rev..

[30] Dogan Ö., Babiz H., Gözen A.G., Budak S. (2011). Synthesis of 2-aziridinyl phosphonates by modified Gabriel–Cromwell reaction and their antibacterial activities. Eur. J. Med. Chem..

[31] Chebanov V.A., Zbruyev A.I., Desenko S.M., Orlov V.D., Yaremenko F.G. (2008). Three-Membered Azaheterocycles Based on α, β-Unsaturated Ketones. Curr. Org. Chem..

[32] Singh G.S., D’hooghe M., De Kimpe N. (2007). Synthesis and Reactivity of C-Heteroatom-Substituted Aziridines. Chem. Rev..

[33] Callebaut G., Meiresonne T., De Kimpe N., Mangelinckx S. (2014). Synthesis and Reactivity of 2-(Carboxymethyl)aziridine Derivatives. Chem. Rev..

[34] Li X., Chen N., Xu J. (2010). An improved and mild Wenker synthesis of aziridines. Synthesis.

[35] Fishbein P.L., Kohn H. (1987). Synthesis and antineoplastic activity of 1a-formyl and 1a-thioformyl derivatives of mitomycin C and 2-methylaziridine. J. Med. Chem..

[36] Kwan B.W., Chowdhury N., Wood T.K. (2015). Combatting bacterial infections by killing persister cells with mitomycin C. Environ. Microbiol..

[37] Higashi Y., Tokushige M., Umezawa H. (1988). Specific inhibition of aspartase by *S*-2,3-dicarboxyaziridine. Biochem. Int..

